# Daytime Napping and the Risk of All-Cause and Cause-Specific Mortality: A 13-Year Follow-up of a British Population

**DOI:** 10.1093/aje/kwu036

**Published:** 2014-03-30

**Authors:** Yue Leng, Nick W. J. Wainwright, Francesco P. Cappuccio, Paul G. Surtees, Shabina Hayat, Robert Luben, Carol Brayne, Kay-Tee Khaw

**Keywords:** aging, mortality, napping, respiratory, siesta, sleep, survival analysis

## Abstract

Epidemiologic studies have reported conflicting results on the relationship between daytime napping and mortality risk, and there are few data on the potential association in the British population. We investigated the associations between daytime napping and all-cause or cause-specific mortality in the European Prospective Investigation Into Cancer-Norfolk study, a British population-based cohort study. Among the 16,374 men and women who answered questions on napping habits between 1998 and 2000, a total of 3,251 died during the 13-year follow-up. Daytime napping was associated with an increased risk of all-cause mortality (for napping less than 1 hour per day on average, hazard ratio = 1.14, 95% confidence interval: 1.02, 1.27; for napping 1 hour or longer per day on average, hazard ratio = 1.32, 95% confidence interval: 1.04, 1.68), independent of age, sex, social class, educational level, marital status, employment status, body mass index, physical activity level, smoking status, alcohol intake, depression, self-reported general health, use of hypnotic drugs or other medications, time spent in bed at night, and presence of preexisting health conditions. This association was more pronounced for death from respiratory diseases (for napping less than 1 hour, hazard ratio = 1.40, 95% confidence interval: 0.95, 2.05; for napping 1 hour or more, hazard ratio = 2.56, 95% confidence interval: 1.34, 4.86) and in individuals 65 years of age or younger. Excessive daytime napping might be a useful marker of underlying health risk, particularly of respiratory problems, especially among those 65 years of age or younger. Further research is required to clarify the nature of the observed association.

In recent years, there has been growing evidence of a relationship between habitual sleep and the risk of mortality from all-causes and cardiovascular diseases (CVDs) (1–3). Most of these studies focused on nighttime sleep duration (4, 5); the implications of daytime napping are poorly understood. A long-established practice in Mediterranean areas, daytime napping, namely siesta, is often linked with good health through a postulated “stress relief” mechanism (6). However, daytime sleepiness, often characterized by daytime napping, has been suggested as an early sign or risk indicator of a range of health problems (7–9). To date, the association between daytime napping and mortality risk is uncertain.

Most previous studies have come from Mediterranean countries (10–13), in which the incidence of and rate of death from CVD tend to be low (6). Several prospective cohort studies from Israel showed that an increased mortality rate was associated with siesta among older people (10, 11), whereas 1 study from Greece suggested that the siesta is protective against heart-related deaths, especially in working men (12). In addition, conflicting United States–based research has found an increased risk of death associated with daytime napping in both men only (14) and women only (15). In both studies, only naps of long durations were found to be significant. A Japanese study suggested that daytime napping was associated with increased risks of all-cause and CVD mortality, but this association was largely explained by comorbid conditions (16). Meanwhile, there is limited evidence from the British population. Although daytime napping has not been a tradition among British adults, the prevalence of napping is likely to increase with the rapidly aging population (17, 18), and it is therefore important to understand the health associations of daytime napping among the British.

Epidemiologic evidence of` the association between daytime napping and mortality is inconsistent and dependent on cultural, environmental, and demographic factors (19). It remains unclear whether napping is beneficial or a risk factor for or marker of ill health. We therefore examined the association between daytime napping and all-cause and cause-specific mortality in a middle- to older-aged British population.

## METHODS

### Participants and measures

Data were drawn from the European Prospective Investigation Into Cancer (EPIC)-Norfolk prospective cohort study. The design and study methods of EPIC-Norfolk have been described previously (20, 21). Briefly, 25,639 men and women aged 40–79 years were recruited using general practice age-sex registers from Norfolk, United Kingdom, and attended a baseline health check during 1993–1997. These participants were then followed up for 2 further health checks from 1996 to 2000 and from 2006 to 2011. In between these health examinations, participants were sent questionnaires for completion and return by post. The Norwich District Ethics Committee approved the study, and all participants gave signed informed consent.

During 1998–2000, a total of 16,374 participants completed the following question in the health questionnaire: “Do you normally take a nap during the day?” If they answered in the affirmative, they were asked to categorize the duration of their nap (<1 hour or ≥1 hour). Napping habits were thereby summarized as: no napping, napping less than 1 hour, and napping 1 hour or more per day.

Covariates measured closer in time to the measurement of napping were chosen and included those measured at baseline or at the time of the second health check (1998–2000). Covariates measured at baseline were social class (professionals, managerial and technical occupations, skilled workers subdivided into nonmanual and manual, partly skilled workers, or unskilled manual workers), educational level (highest qualification attained: no qualifications, educated to age 16 years, educated to age 18 years, or educated to degree level), and physical activity level (inactive, moderately inactive, moderately active, or active) (22). Body mass index (BMI; weight in kilograms divided by height in meters squared) was measured at the baseline health check. Other covariates were reported in questionnaires during the time of the second health check: age, marital status (single, married, widowed, separated, or divorced), employment status (working or not working), smoking status (current, former, or nonsmokers), alcohol intake (units of alcohol drunk per week), self-reported general health (excellent, good, moderate, or poor), use of hypnotic drugs (yes or no), antidepressant use (yes or no), use of drugs to treat chronic obstructive pulmonary disease (COPD) (yes or no), major depressive disorder (MDD) in the previous year (yes or no) (23), and time spent in bed at night (in hours, derived from the differences between reported rise times and bedtimes). Hypnotic drug use was defined according to the British National Formulary (section 4.1) (24) and included benzodiazepines, zaleplon, zolpidem, and zopiclone, chloral and its derivatives, clomethiazole, and antihistamines. Preexisting health conditions included self-reported stroke, myocardial infarction, diabetes, cancer, asthma, bronchitis, and emphysema, as well as a proxy measure of obstructive sleep apnea (OSA), with persons who were in the highest BMI quartile and who reported taking antihypertension drugs being defined as likely to have underlying OSA.

Participants were flagged for death certification at the United Kingdom Office of National Statistics so that we were notified of their deaths, with vital status established for the whole cohort. The current analysis presents deaths that occurred during followed up from January 2000 and until December 2012. Deaths were coded initially according to the *International Classification of Diseases* (ICD), *Ninth Revision*, and later the *Tenth Revision*. The underlying cause of death was categorized as due to CVD (ICD-9 codes 401–448 or ICD-10 codes I10–I79), cancer (ICD-9 codes 140–208 or ICD-10 codes C00–C97), or respiratory diseases (ICD–9 codes 460–496 or ICD-10 codes J00–J99).

### Statistical analysis

The present study included 16,374 participants (7,161 men and 9,213 women) who provided information on their daytime napping habits. The characteristics of the participants were first compared by category of daytime napping. The comparisons of normally distributed variables and skewed continuous variables were based on analysis of variance and Kruskal-Wallis test, respectively. Categorical variables were compared using Pearson's χ^2^ test.

The associations between daytime napping and all-cause or disease-specific mortality were examined using Cox proportional hazards regression models that were adjusted for covariates. The endpoints were grouped into death from all causes, CVD, cancer, respiratory diseases, and all other causes. We firstly examined the proportional hazard assumption using both a Kaplan-Meier plot and Schoenfeld's test, and no sign of violation was found for any endpoint. All covariates were chosen a priori based on the literature and their relevance to napping and health. Models were constructed with progressive adjustment of the covariates to show the associations explained by the covariates: 1) Model A was adjusted for age and sex; 2) model B was further adjusted for social class, educational level, marital status, employment status, BMI, physical activity level, smoking status, and alcohol intake; 3) model C was adjusted for the variables in model B and MDD, self-reported general health, time spent in bed at night, hypnotic drug use, antidepressant use, and COPD drug use; and 4) model D was further adjusted for the variables in all models and preexisting health conditions. Each multivariable model was built on persons for whom we had complete measurements of all covariates.

Finally, we performed stratified analysis with adjustment for all the covariates according to follow-up length and preexisting health conditions and according to potential effect modifiers: age (≤65 vs. >65 years old), sex, smoking status (ever vs. never smoker), employment status, BMI (dichotomized using the median), MDD, time spent in bed at night (dichotomized using the median), and nighttime sleep duration (reported in hours at the time of the third health check). Hazard ratios (with 95% confidence intervals) were calculated using no napping as the reference group (with adjustment for sex through inclusion of sex-specific baseline hazards). The overall associations between the 2 categories of napping and mortality were tested using the likelihood ratio test. All statistical tests were 2-sided, and a *P* value <0.05 was considered statistically significant. Analyses were implemented in Stata, version 12.0 (StataCorp LP, College Station, Texas).

## RESULTS

During 1998–2000, napping during the day was reported by 2,653 of 7,161 men (37.0%) and 2,228 of 9,213 women (24.2%). Tables 1 and 2 show participants' baseline characteristics by napping habits. Among the persons who took naps, only 10% napped for more than 1 hour per day. Those who took naps were more likely to be men and were on average 6 years older than those who did not nap. Persons who took naps were more likely to be smokers, not working, and less active and to have a lower educational level, higher BMI, and poor self-reported general health. The above associations were all stronger for those who napped for longer time periods.

**Table 1.** Baseline Characteristics by Categories of Napping Behaviors in 7,161 Men, European Prospective Investigation Into Cancer-Norfolk, United Kingdom, 1998–2000

Characteristic	Total No.	Time Spent Napping					
		None		<1 hour		≥1 hour	
		No.	%	No.	%	No.	%
All men^a^	7,161	4,508^b^	63.0	2,379	33.2	274	3.8
Age, years							
≤65	4,125	3,072^b^	68.2	942	39.7	111	40.5
>65	3,027	1,431	31.8	1,433	60.3	163	59.5
Social class							
Nonmanual worker	4,319	2,741	61.6	1,425	60.9	153	56.7
Manual worker	2,738	1,708	38.4	913	39.1	117	43.3
Education category							
Lower	2,650	1,531^b^	34.0	989	41.6	130	47.4
Higher	4,509	2,976	66.0	1,389	58.4	144	52.6
Marital status							
Single	303	193	4.3	97	4.1	13	4.7
Married	6,244	3,936	87.6	2,075	87.6	233	85.0
Other^c^	588	362	8.1	198	8.4	28	10.2
Smoking status							
Current smoker	608	362^b^	8.1	203	8.6	43	15.8
Former smoker	3,980	2,378	53.2	1,445	61.2	157	57.5
Never smoker	2,517	1,730	38.7	714	30.2	73	26.7
Category of alcohol intake							
Lower	3,287	2,063	50.8	1,107	52.3	117	51.1
Higher	3,120	1,998	49.2	1,010	47.7	112	48.9
Category of body mass index^d^							
Lower (<25.7)	3,201	2,142^b^	52.8	966	45.9	93	39.9
Higher (≥25.7)	3,189	1,911	47.2	1,138	54.1	140	60.1
Physical activity level							
Inactive/moderately inactive	3,834	2,295^b^	50.9	1,354	56.9	185	67.5
Moderately active/active	3,327	2,213	49.0	1,025	43.1	89	32.5
Major depressive disorder							
No	5,976	4,448^b^	98.7	2,341	98.4	266	97.1
Yes	231	60	1.3	38	1.6	8	2.9
Self-reported general health							
Excellent	1,150	805^b^	18.0	323	13.6	22	8.1
Good	4,732	3,009	67.3	1,579	66.6	144	53.1
Poor to moderate	1,233	660	14.8	468	19.8	105	38.7
Hypnotic drug use							
No	7,055	4,448	98.7	2,341	98.4	266	97.1
Yes	106	60	1.3	38	1.6	8	2.9
Time in bed per night, hours							
≤8.5	3,611	2,333^e^	60.4	1,167	57.5	111	52.6
>8.5	2,493	1,532	39.6	861	42.5	100	47.4
Preexisting health conditions^f^							
No	4,505	3,104^b^	73.1	1,285	59.7	116	47.0
Yes	2,142	1,144	26.9	867	40.3	131	53.0

^a^ Comparison was made between men and women.

^b^ *P* < 0.001.

^c^ Widowed, separated, or divorced.

^d^ Weight (kg)/height (m)^2^.

^e^ *P* < 0.05.

^f^ Stroke, myocardial infarction, cancer, asthma, bronchitis, and underlying sleep apnea.

**Table 2.** Baseline Characteristics by Categories of Napping Behaviors in 9,213 Women, European Prospective Investigation Into Cancer-Norfolk, United Kingdom, 1998–2000

Characteristic	Total No.	Time Spent Napping					
		None		<1 hour		≥1 hour	
		No.	%	No.	%	No.	%
All women^a^	9,213	6,985	75.8	2,033	22.1	195	2.1
Age, years							
≤65	5,820	4,888^b^	70.1	840	41.4	92	47.2
>65	3,378	2,084	29.9	1,191	58.6	103	52.8
Social class							
Nonmanual worker	5,755	4,404	64.3	1,230	62.2	121	64.4
Manual worker	3,264	2,450	35.7	747	37.8	67	35.6
Education category							
Lower	4,687	3,427^b^	49.1	1,146	56.4	114	58.5
Higher	4,521	3,553	50.9	887	43.6	81	41.5
Marital status							
Single	387	280^b^	4.0	95	4.7	12	6.2
Married	6,750	5,236	75.4	1,389	68.5	125	64.4
Other^c^	2,030	1,430	20.6	543	26.8	57	29.4
Smoking status							
Current smoker	769	596^b^	8.6	142	7.1	31	16.0
Former smoker	2,997	2,205	31.9	727	36.1	65	33.5
Never smoker	5,352	4,110	59.5	1,144	56.8	98	50.5
Category of alcohol intake							
Lower	4,601	3,484^d^	59.9	1,027	63.0	90	66.2
Higher	2,984	2,336	40.1	602	37.0	46	33.8
Category of body mass index^e^							
Lower (<25.7)	4,096	3,250^b^	52.0	766	43.6	80	48.5
Higher (≥25.7)	4,071	2,995	48.0	991	56.4	85	51.5
Physical activity level							
Inactive/moderately inactive	5,551	4,092^b^	58.6	1,324	65.1	135	69.3
Moderately active/active	3,662	2,893	41.4	709	30.9	60	30.8
Major depressive disorder							
No	7,458	5,656	93.6	1,650	94.3	153	90.5
Yes	501	385	6.4	100	5.7	16	9.5
Self-reported general health							
Excellent	1,419	1,174^b^	16.9	225	11.1	20	10.4
Good	5,998	4,600	66.3	1,304	64.6	94	49.0
Poor to moderate	1,853	1,166	16.8	490	24.3	78	40.6
Hypnotic drug use							
No	9,042	6,871^d^	98.4	1,983	97.5	188	96.4
Yes	171	114	1.6	50	2.5	7	3.6
Time in bed per night, hours							
≤8.5	3,550	2,725^d^	45.2	774	45.7	51	33.6
>8.5	4,321	3,299	54.8	921	54.3	101	66.4
Preexisting health conditions^f^							
No	5,307	4,192^b^	66.9	1,015	59.4	100	63.3
Yes	2,827	2,076	33.1	693	40.6	58	36.7

^a^ Comparison was made between men and women.

^b^ *P* < 0.001.

^c^ Widowed, separated, or divorced.

^d^ *P* < 0.05.

^e^ Weight (kg)/height (m)^2^.

^f^ Stroke, myocardial infarction, cancer, asthma, bronchitis, and underlying sleep apnea.

A total of 3,251 deaths (1,034 from CVD, 1,213 from cancer, 286 from respiratory diseases, and 718 from all other causes) were observed during the 13-year follow-up. There were 1,795 deaths in men and 1,456 in women. Table 3 shows the adjusted hazard ratios associated with napping. After adjustment for age and sex, daytime napping was associated with a 22% increase in the risk of all-cause mortality in persons who napped less than 1 hour (hazard ratio (HR) = 1.22, 95% confidence interval (CI): 1.13, 1.31) and a 54% increase for persons who napped 1 hour or longer (HR = 1.54, 95% CI: 1.31, 1.81). The hazard ratios were attenuated to 1.14 (95% CI: 1.03, 1.27) for napping less than 1 hour and 1.34 (95% CI: 1.07, 1.68) for napping 1 hour or longer after further adjustment for social class, educational level, marital status, employment status, BMI, physical activity level, smoking status, alcohol intake, MDD, self-reported general health, hypnotic drug use, antidepressant and COPD drug use, and time spent in bed at night. This association was little changed after additional adjustment for preexisting health conditions, with a 14% and 32% increase in all-cause mortality risk among those who napped for less than 1 hour or 1 hour or more per day, respectively. This association was more pronounced for death from respiratory diseases (for napping <1 hour, HR = 1.40, 95% CI: 0.95, 2.05; for napping ≥1 hour, HR = 2.56, 95% CI: 1.34, 4.86). No statistically significant associations were observed for death from other causes after full adjustments.

**Table 3.** Associations Between Daytime Napping and All-Cause and Cause-Specific Mortality in 16,374 Men and Women, European Prospective Investigation Into Cancer-Norfolk, United Kingdom, 2000–2012

Time Spent Napping by Cause and No. of Deaths	Total No.	Model A^a^		Model B^b^		Model C^c^		Model D^d^	
		HR	95% CI	HR	95% CI	HR	95% CI	HR	95% CI
No napping	11,493	1.00	Referent	1.00	Referent	1.00	Referent	1.00	Referent
All-cause mortality (*n* = 3,251)									
<1 hour	4,412	1.22^e^	1.13, 1.31	1.25^e^	1.15, 1.37	1.14^f^	1.03, 1.27	1.14^g^	1.02, 1.27
≥1 hour	469	1.54^e^	1.31, 1.81	1.56^e^	1.30, 1.88	1.34^g^	1.07, 1.68	1.32^g^	1.04, 1.68
Overall *P*		<0.001		<0.001		<0.01		0.01	
Cardiovascular disease (*n* = 1,034)									
<1 hour	4,412	1.24^f^	1.09, 1.41	1.22^g^	1.05, 1.43	1.05	0.88, 1.26	1.00	0.81, 1.22
≥1 hour	469	1.70^e^	1.30, 2.21	1.59^f^	1.16, 2.19	1.03	0.67, 1.57	0.92	0.57, 1.48
Overall *P*		<0.001		0.003		0.86		0.94	
Cancer (*n* = 1,213)									
<1 hour	4,412	1.17^g^	1.04, 1.33	1.17^g^	1.01, 1.34	1.15	0.97, 1.35	1.18	1.00, 1.41
≥1 hour	469	1.10	0.81, 1.50	1.15	0.81, 1.63	1.27	0.86, 1.88	1.27	0.84, 1.92
Overall *P*		0.04		0.09		0.18		0.12	
Respiratory disease (*n* = 286)									
<1 hour	4,412	1.36^g^	1.06, 1.74	1.65^e^	1.23, 2.23	1.45^g^	1.01, 2.07	1.40	0.95, 2.05
≥1 hour	469	2.3^e^	1.48, 3.65	2.81^e^	1.66, 4.75	2.51^f^	1.37, 4.60	2.56^f^	1.34, 4.86
Overall *P*		0.001		<0.001		<0.01		0.02	
All other causes (*n* = 718)									
<1 hour	4,412	1.22^g^	1.04, 1.43	1.31^f^	1.09, 1.58	1.18	0.95, 1.45	1.18	0.94, 1.47
≥1 hour	469	1.68^f^	1.21, 2.33	1.75^f^	1.18, 2.59	1.46	0.92, 2.32	1.56	0.96, 2.53
Overall *P*		<0.01		<0.01		0.15		0.13	

Abbreviations: CI, confidence interval; HR, hazard ratio.

^a^ Adjusted for age and sex.

^b^ Adjusted for the variables in model A and social class, educational level, marital status, employment status, body mass index (weight (kg)/height (m)^2^), physical activity level, smoking status, and alcohol intake.

^c^ Adjusted for the variables in model B and depression, self-reported general health, hypnotic drug use, antidepressant use, chronic obstructive pulmonary disease drug use, and time spent in bed at night.

^d^ Adjusted for the variables in model C and self-reported preexisting diseases and underlying sleep apnea.

^e^ *P* < 0.001.

^f^ *P* < 0.01.

^g^ *P* < 0.05.

Table 4 shows results from stratified analysis for all-cause mortality. The association was stronger in those who were younger (for napping <1 hour, HR = 1.42, 95% CI: 1.13, 1.79; for napping ≥1 hour, HR = 1.95, 95% CI: 1.16, 3.28; *P* for interaction = 0.04 for persons 42–65 years of age compared with those 65–82 years of age) and in those who had a lower BMI (for napping <1 hour, HR = 1.32, 95% CI: 1.12, 1.56; for napping ≥1 hour, HR = 1.72, 95% CI: 1.21, 2.45; *P* for interaction = 0.02 for those with a BMI of 15.2–25.7 compared with those with a BMI of higher than 25.7). In addition, there was a suggestion that this association was stronger in persons who reported experiencing MDD in the past 12 months (for napping ≥1 hour, HR = 2.90, 95% CI: 1.12, 7.50; *P* for interaction = 0.13) and in those who were working (for napping ≥1 hour, HR = 2.46, 95% CI: 1.16, 5.20; *P* for interaction = 0.33). No statistically significant differences were observed by sex, cigarette smoking history, time spent in bed at night, nighttime sleep duration, or preexisting health conditions (among those who reported no preexisting CVD, cancer, asthma, bronchitis, or underlying sleep apnea at baseline and who napped for ≥1 hour, HR = 1.41, 95% CI: 1.03, 1.93). Furthermore, there was no evidence that the association attenuated with increasing length of follow-up (in these data, the association was stronger after 6.5 years of follow-up; *P* for interaction = 0.07).

**Table 4.** Multivariable^a^ Associations Between Daytime Napping and All-Cause Mortality by Subgroups, European Prospective Investigation Into Cancer-Norfolk, United Kingdom, 2000–2012

Variable	No. of Deaths	Time Spent Napping^b^			
		<1 hour		≥1 hour	
		HR	95% CI	HR	95% CI
Length of follow-up, years					
<6.5	1,189	0.89	0.73, 1.08	1.06	0.69, 1.63
≥6.5	2,062	1.10	0.97, 1.26	1.41^c^	1.05, 1.89
*P* for interaction		0.07			
Age, years					
≤65	783	1.42^d^	1.13, 1.79	1.95^c^	1.16, 3.28
>65	2,464	1.05	0.93, 1.19	1.19	0.90, 1.56
*P* for interaction		0.04			
Sex					
Men	1,795	1.1	0.96, 1.26	1.3	0.98, 1.71
Women	1,456	1.23^c^	1.02, 1.47	1.19	0.72, 1.98
*P* for interaction		0.70			
Employment status					
Working	422	1.23	0.90, 1.67	2.46^c^	1.16, 5.20
Not working	2,679	1.13^c^	1.00, 1.27	1.23	0.96, 1.59
*P* for interaction		0.32			
Preexisting health conditions^e^					
Yes	1,431	1.22^c^	1.03, 1.45	1.23	0.85, 1.77
No	1,820	1.08	0.94, 1.24	1.44^c^	1.04, 1.99
*P* for interaction		0.93			
Smoking status					
Current or former smoker	2,042	1.19^c^	1.04, 1.36	1.22	0.91, 1.63
Never smoked	1,163	1.06	0.88, 1.28	1.54	0.99, 2.37
*P* for interaction		0.90			
Category of body mass index^f^					
Lower (<25.7)	1,393	1.32^g^	1.12, 1.56	1.72^d^	1.21, 2.45
Higher (≥25.7)	1,848	1.04	0.90, 1.20	1.08	0.77, 1.50
*P* for interaction		0.02			
Major depressive disorder					
Yes	136	1.52	0.79, 2.96	2.90^c^	1.12, 7.50
No	2,702	1.13^c^	1.02, 1.27	1.25	0.97, 1.62
*P* for interaction		0.13			
Time spent in bed at night, hours					
≤8.5	1,234	1.08	0.93, 1.26	1.54^c^	1.10, 2.16
>8.5	1,427	1.20^c^	1.03, 1.40	1.13	0.80, 1.61
*P* for interaction		0.78			
Nighttime sleep duration, hours^h^					
<6	305	1.10	0.76, 1.60	1.64	0.69, 3.94
6–7	234	0.88	0.58, 1.32	2.02	0.78, 5.23
>7	362	1.13	0.84, 1.52	0.82	0.39, 1.74
*P* for interaction		0.31			

Abbreviations: CI, confidence interval; HR, hazard ratio.

^a^ Adjusted for age, sex, social class, educational level, marital status, employment status, body mass index, physical activity level, smoking status, alcohol intake, major depressive disorder, self-reported general health, hypnotic drug use, antidepressant use, chronic obstructive pulmonary disease drug use, time spent in bed at night, and preexisting health conditions.

^b^ The reference group was persons who did not nap.

^c^ *P* < 0.05.

^d^ *P* < 0.01.

^e^ Stroke, myocardial infarction, cancer, asthma, bronchitis, emphysema, and underlying sleep apnea.

^f^ Weight (kg)/height (m)^2^.

^g^ *P* < 0.001.

^h^ Measured in the third health check in 10,520 people; the others were all measured in the second health check.

For respiratory deaths, the associations in current and former smokers (for napping ≥1 hour, HR = 2.32, 95% CI: 1.10, 4.90) were similar to those in never smokers (for napping ≥1 hour, HR = 3.59, 95% CI: 0.95, 13.58; *P* for interaction = 0.49). In addition, no statistically significant differences were observed according to age, sex, employment status, smoking status, COPD drug use, forced expiratory volume in 1 second, or preexisting asthma, bronchitis, or emphysema.

## DISCUSSION

In the present large sample of middle- to older-aged British adults, daytime napping of 1 hour per day or more was associated with a 32% increase in the risk all-cause mortality during the 13-year follow-up. This association was independent of age, sex, social class, educational level, marital status, employment status, BMI, physical activity level, smoking status, alcohol intake, MDD, self-reported general health, time spent in bed at night, hypnotic drug use, antidepressant use, COPD drug use, and preexisting health conditions and was similar among men and women. The associations were more pronounced for deaths from respiratory illness and for participants who were 65 years of age or younger. Furthermore, the association between napping and all-cause mortality remained among those without preexisting health conditions and did not attenuate with increasing length of follow-up.

To our knowledge, this is the first study to present in detail the association between daytime napping and cause-specific mortality in a British population. Strengths of our study include the long follow-up and range of measures for which we were able to adjust. Our study has several limitations. First, 16,374 of the 25,639 participants at baseline completed the subsequent mailed questionnaire that contained the napping question. Compared with the rest of the participants, these 16,374 people were more likely to be women and to have a higher social class and educational level. Therefore, our results may not be generalizable to populations with lower socioeconomic status. Second, the information on napping was obtained from self-report. Previous studies (25, 26) have shown a tendency for older people to underreport napping behaviors, and this may lead to underestimation of the association. It was suggested that naps of less than 30 minutes (usually known as power naps) can be largely beneficial, especially among shift workers (27–29), whereas naps longer than 30 minutes can cause sleep inertia and are usually not recommended (30). Napping duration was categorized into less than 1 hour and 1 hour or more in our questionnaire, and we were therefore unable to differentiate the effects of power naps from those of excessive napping. Although this might have diluted the overall association, we conclude from the current study that there is an increased health risk associated with excessive daytime napping.

Voluntary naps and naps as a result of underlying pathology have different implications for health, and identification of the reasons for the naps is crucial. Although a range of preexisting health conditions and medication use was considered in this study, we cannot rule out that our results may be partly explained by the effects of other undiagnosed health problems or medications not included in this study that might cause daytime fatigue or sleepiness. Daytime napping might be a consequence of nighttime sleep disturbance, which has been associated with an increased risk of death (31). Although the detailed purpose of napping was not recorded in the present study, the association between daytime napping and mortality risk did not change by nighttime sleep durations and was independent of time spent in bed at night, which has been suggested to be a good indicator of nighttime sleep patterns in the EPIC-Norfolk population (32). In addition, a surrogate measure of OSA was included in regression models to help address the problem. This measure combines BMI and hypertension, 2 strong correlates of OSA (33–35). The surrogate measure may partly reflect the probability of having OSA; therefore, further studies with more precise measures of OSA and nighttime sleep quality are required to confirm whether the association stands independent of other sleep parameters.

Our findings are consistent with most previous studies that have reported an association between self-reported daytime napping and increased all-cause mortality (10, 11, 14–16, 36, 37). These studies covered populations from the Mediterranean, Japan, and the United States and included primarily older populations. The largest published report to date came from the Japan Collaborative Cohort Study, in which a total of 9,643 deaths were recorded and daytime napping was found to be associated with increased mortality from specific causes, particularly CVD (16). Indeed, most previous studies have related daytime napping to increased CVD mortality risk (10, 15, 16, 37). In our study, the association between napping and CVD mortality risk was largely explained by MDD in the previous year. MDD is commonly associated with sleep disturbance, which might lead to excessive daytime napping. Previous evidence based upon our cohort (23) has shown major depression to be associated with an increased risk of ischemic heart disease mortality, though understanding of this association remains elusive. It is possible that the association between napping and CVD mortality is mostly explained by depression or MDD reported within a certain time frame. Future studies would need to carefully examine the role of depression in the napping-mortality association.

At the same time, several earlier studies (6, 12) from Mediterranean populations have suggested different findings. The Greek EPIC cohort study, which is methodologically comparable to our study, found napping to be protective against coronary mortality (12). Notably, the Greek cohort included people who were 20–86 years of age at baseline, whereas our participants were exclusively middle- to older-aged adults. Moreover, the association was particularly strong among working men in their study, which might be explained by the possible stress-releasing effect of afternoon naps (12). This effect could have been masked in our aging population, who are mostly retired and might take naps for other purposes, such as compensating for declining physical functions. Interestingly, in our study, the positive association between napping and mortality was seen to be stronger among persons 65 years of age or younger than among those older than 65 years of age (65 years is the age by which most people have retired from work in the United Kingdom). It is often believed that napping is protective for younger populations and problematic for older ones (12, 19, 37). Notably, the average age of our participants at baseline was 62 years, so the younger persons in the present study were not comparable to those mentioned in earlier studies (12, 38). Although it is unknown why the association was stronger among those 65 years of age or younger, our finding raises the possibility that napping might be more indicative of mortality risk in this population, and this needs to be confirmed by further studies. Unlike in previous studies (14, 36), no evident sex difference was observed in this study.

In the present study, we found large effect sizes for the association between napping and death from respiratory disease even though there were much fewer respiratory deaths than CVD and cancer deaths. The association was independent of smoking status, preexisting respiratory diseases, and COPD drug use. This is surprising given the lack of evidence of the associations between napping and respiratory health. Although none of the previous studies highlighted the association between napping and respiratory mortality, most of them did suggest the association with non-CVD/non-cancer mortality (10, 15, 16), and these categories were largely made up of respiratory mortality in our study. The reason why daytime napping may increase respiratory mortality is not known, but it is worth noting the interrelationship between the cardiovascular and respiratory systems (39). Although we failed to find an association between napping and CVD mortality, the napping-respiratory association might not be separate from the cardiovascular consequences of sleep problems (e.g., OSA). It seems reasonable to suggest that persons with respiratory problems (especially hypoxic conditions) were more likely to nap and were also more likely to die of respiratory disease because of their underlying problems. The incidence of OSA was not recorded directly in this study, and a proxy measure was used to account for the effects. Given the close link between OSA and mortality (40, 41), daytime napping might well be a good surrogate measure of OSA, which is potentially important for early detection of OSA in the general population.

Although in this study the association between napping and all-cause mortality remained after excluding participants with known preexisting health conditions at baseline, it remains possible that daytime napping is simply a marker of undiagnosed health problems and of reverse causation, with those individuals who have undiagnosed ill health napping more. In this case, we would expect attenuation in the association whereby it is stronger in the immediate period of follow-up and lessens thereafter. However, the association seemed to be even stronger among those with a follow-up length of 6.5 years or more. Interestingly, the association was stronger among persons with a lower BMI. Although the exact reason for this observation is unclear, it is worth noting the “obesity paradox,” which might have been brought about by existing physiological or pathological changes (42, 43). It is possible that persons with lower BMI could have been suffering from underlying illnesses that led to weight loss and that this added to the health risk conveyed by daytime napping.

In the United Kingdom, daytime napping is not part of the cultural norm, and in the absence of obvious disruptions in nighttime sleep patterns, it remains plausible that napping might be an early sign of system disregulation and a marker of future health problems. Alternatively, there may be a biological mechanism through which napping is associated with mortality, for example, through inflammatory pathways, especially increased chronic low-grade inflammation. Increased levels of inflammation have been suggested for persons with reduced nighttime sleep durations (44), but we are unaware of any evidence to show an association between daytime napping and inflammation. Our findings need confirmation from further population studies that include examination of the relationship between napping and physiological biomarkers. This further work could help evaluate whether there is a causal link between napping and mortality risk or whether napping is a marker of system disregulation or of undiagnosed respiratory problems that lead to an increased risk of future ill health.

In summary, independent associations were found between daytime napping and an increased risk of mortality from all causes and respiratory diseases in a large population-based middle- to older-aged British cohort. The association remained after exclusion of persons who died within the first 6.5 years of follow-up and was more pronounced for people 65 years of age or younger. The exact mechanisms of these associations remain unknown. Further studies are needed before any recommendations can be made. Excessive daytime napping might be a useful marker of underlying health risks, particularly respiratory problems, especially among those 65 years of age or younger.
